# New insight into long non-coding RNAs associated with bone metastasis of breast cancer based on an integrated analysis

**DOI:** 10.1186/s12935-021-02068-7

**Published:** 2021-07-13

**Authors:** Yu Zhang, Xiaofeng Huang, Jin Liu, Guo Chen, Chengjun Liu, Sen Zhang, Jiaxin Li

**Affiliations:** 1 Department of Orthopaedics, The First People’s Hospital of Chengdu, Chengdu, Sichuan Province China; 2 State Key Laboratory of Bioactive Substances and Functions of Natural Medicines, Institute of Materia Medica, Chinese Academy of Medical Sciences & Peking Union Medical College, Beijing, 100050 People’s Republic of China; 3 Department of Gastrointestinal Surgery, The Sixth Affiliated Hospital of Wenzhou Medical University & Lishui City People’s Hospital, Lishui, Zhejiang Province China

**Keywords:** Breast cancer, Bone metastasis, lncRNA, GEO, Interaction pairs, LOC641518, LEF1

## Abstract

**Background:**

Bone is the most common site of metastatic breast cancer, and it is a leading cause of breast cancer-related death. This study aimed to explore bone metastasis-related long non-coding RNAs (lncRNAs) in breast cancer.

**Methods:**

Four mRNA datasets and two lncRNA datasets of bone metastasis, lung metastasis and liver metastasis of breast cancer were downloaded from Gene Expression Omnibus (GEO) database. The differentially expressed mRNAs (DEmRNAs) and lncRNAs (DElncRNAs) in group of bone metastasis vs lung metastasis and bone metastasis vs liver metastasis, as well as the overlap of the two groups, were identified. Gene ontology (GO) and Kyoto Encyclopedia of Genes and Genomes (KEGG) pathway analysis and protein–protein interaction (PPI) network construction of DEmRNAs were conducted. The *cis* nearby-targeted DEmRNAs of DElncRNAs were obtained. Quantitative real-time polymerase chain reactions (qRT-PCR) was used to detect the expression levels of selected DEmRNAs and DElncRNAs. LOC641518-lymphoid enhancer-binding factor 1 (LEF1) pair was selected to verify its role in migration and invasion capability of breast cancer cells by wounding healing assay and transwell invasion assay.

**Results:**

A total of 237 DEmRNAs were obtained in bone metastasis compared with both lung metastasis and liver metastasis. A total of three DElncRNAs in bone metastasis compared with both lung metastasis and liver metastasis were obtained. A total of seven DElncRNA-nearby-targeted DEmRNA pairs and 15 DElncRNA-nearby-targeted DEmRNA pairs in group of bone metastasis vs lung metastasis and bone metastasis vs liver metastasis, were detected, respectively. Four cis LncRNA-mRNA interaction pairs were identified, which are LOC641518-LEF1, FLJ35024-Very Low Density Lipoprotein Receptor (VLDLR), LOC285972-Retinoic Acid Receptor Responder 2 (RARRES2) and LOC254896-TNF receptor superfamily member 10c (TNFRSF10C). qRT-PCR using clinical samples from our hospital confirms the bioinformatics prediction. siRNA knocking down LOC641518 down-regulates LEF1 mRNA expression, and reduces the migration and invasion capability of breast cancer cells.

**Conclusions:**

We concluded that four LncRNA-mRNA pairs, including LOC641518-LEF1, may play a central role in breast cancer bone metastasis.

**Supplementary Information:**

The online version contains supplementary material available at 10.1186/s12935-021-02068-7.

## Introduction

Globally, breast cancer is the most common malignancy in women [1]. Bone is the most common site of metastatic breast cancer, accounting for nearly three-quarters of patients with metastatic breast cancer [2]. Bone metastasis is often accompanied by bone pain and skeletal-related events, leading to increased mortality and seriously affecting the quality of life of patients [3]. The clinical complications of breast cancer bone metastasis bring heavy burden to the individual and society [4]. Bone metastases secondary to breast cancer are incurable, and the molecular mechanisms are complex and involved in multiple process [4].

Over the past few decades, a great deal of information about lncRNAs has been generated, which facilitated the studies on diverse cancer etiology, including breast cancer [5]. Increasing evidence demonstrated that lncRNAs participate in many cellular process, such as cell fate decision, immune response, cancer cells proliferation and metastasis [6]. Gooding et al. indicated that the lncRNA BORG drives breast cancer metastasis to lung and disease recurrence [7]. Metastasis associated lung adenocarcinoma transcript 1 (MALAT1), previously described as a metastasis-promoting lncRNA, was reported to suppress breast cancer metastasis [8]. HOXA11-AS was demonstrated to promote breast cancer invasion and lymph node metastasis through affecting epithelial-mesenchymal transition [9]. However, there are few studies on bone metastasis-related lncRNAs in breast cancer. Li et al. reported that lncRNA MAYA (MST1/2-Antagonizing for YAP Activation) promoted breast cancer bone metastasis by activating ROR1-HER3 and Hippo-YAP pathways, which indicated important role of lncRNA in breast cancer bone metastasis [10]. Currently, there is also lack of systemic LncRNA-mRNA network analysis based on human database, so it is necessary to explore important lncRNAs associated with breast cancer bone metastasis.

In this present study, a comprehensive integrated analysis of lncRNAs and mRNAs expression profiles of bone metastasis, lung metastasis and liver metastasis of breast cancer downloaded from GEO database were performed. The differentially expressed mRNAs (DEmRNAs) and lncRNAs (DElncRNAs) were acquired. The *cis* nearby-targeted DEmRNAs of DElncRNAs were obtained as well. Finally, we established in vitro cell based assay to investigate and confirm the role of novel LncRNA-mRNA axis in tumor metastasis. This study aimed to provide additional knowledge on the molecular mechanisms of breast cancer bone metastasis, especially the role of pro-bone metastasis LncRNAs.

## Materials and methods

### Dataset collection

To acquire the mRNA and lncRNA expression profiles of bone metastasis, lung metastasis and liver metastasis of breast cancer tissues, datasets in GEO database with the following criteria were retrieved: datasets should be whole-genome mRNA/lncRNA expression profile by array; these data were derived from bone metastasis, lung metastasis and liver metastasis of breast cancer tissues; datasets were normalized or original. After screening, four mRNA datasets and two lncRNA datasets were enrolled in this study and shown in Table 1.

**Table 1:** List of mRNA/lncRNA study samples from GEO database

GEO accession	Platforms	Liver metastasis	Lung metastasis	Bone metastasis	Year	country
mRNA						
GSE14020	GPL96 [HG-U133A] Affymetrix human genome U133A array	5	16	8	2009	USA
	GPL570[HG-U133_Plus_2] Affymetrix human genome U133 plus 2.0 array	0	4	10		
GSE54323	GPL570[HG-U133_Plus_2] Affymetrix human genome U133 plus 2.0 array	7	0	14	2015	Sweden
GSE46141	GPL10379 Rosetta/Merck human RSTA custom Affymetrix 2.0 microarray [HuRSTA-2a520709]	16	2	5	2013	Sweden
lncRNA						
GSE14020	GPL570[HG-U133_Plus_2] Affymetrix human genome U133 plus 2.0 array	0	4	10	2009	USA
GSE54323	GPL570[HG-U133_Plus_2] Affymetrix human genome U133 plus 2.0 array	7	0	14	2015	Sweden

### Differential expression analysis

MetaMA [11] and limma [12] package in R were utilized to acquire the DEmRNAs and DElncRNAs. *P*-values were calculated using a significance threshold of *p*-value < 0.05. With R package “pheatmap”, hierarchical clustering analysis of DEmRNAs and DElncRNAs were performed.

### GO and pathway analysis of DEmRNAs

GeneCodis3 (http://genecodis.cnb.csic.es/analysis) was applied to perform GO and KEGG pathway enrichment analysis of DEmRNAs. The threshold was set as false discover rate (FDR) < 0.05.

### Protein–protein interaction (PPI) network construction

Top 50 up- and down-regulated DEmRNAs were searched with the BioGrid (http://www.uniprot.org/database/DB-0184), and PPI network was constructed with Cytoscape software (version 3.6.1, http://www.cytoscape.org).

### *Cis* nearby-targeted DEmRNAs of the DElncRNAs

DEmRNAs transcribed within a 100-kb window upstream or downstream of DElncRNAs were searched, which were defined as *cis* nearby-targeted DEmRNAs of DElncRNAs, to obtain the targeted DEmRNAs of DElncRNAs with *cis*-regulatory effects.

### Quantitative real-time polymerase chain reaction (qRT-PCR) validation

Twenty-five patients with bone metastasis of breast cancer, 25 patients with lung metastasis of breast cancer, and 25 patients with liver metastasis of breast cancer were incorporated in our study. Meanwhile, 25 primary breast cancer patients without metastasis were enrolled, and their adjacent non-cancer breast tissues was considered as normal tissue to be used as control in the present study. Their tumor tissues from metastatic sites were used for RNA isolation. Ethical approval was obtained from the ethics committee of the First People’s Hospital of Chengdu and informed written consent was obtained from all of subjects. All the patients in the current study were same as described in the previous publication [13].

Total RNA of the tumor tissues from metastatic sites was extracted using the RNA liquid Reagent (TIANGEN Bio, Beijing, China) according to the manufacturer’s protocols. One microgram RNA was applied to synthesize cDNA by Fast Quant Reverse Transcriptase (TIANGEN Bio). Then real-time PCR was performed in an ABI 7300 Real-time PCR system with SYBR R Green PCR MasterMix (TIANGEN). All reactions were carried out in triplicate. GAPDH was used for the internal reference. Relative gene expression was analyzed by fold change method. And all the primer sequence was shown in Additional file 1: Table S1.

### Cell culture and transfection with plasmid and siRNA

Human breast cancer cell line MCF-7 and MDA-MB-231, and non-carcinoma human breast epithelial cell line MCF-10A were purchased from ATCC (Manassas, VA, USA) and were cultured in RPMI 1640 medium (Invitrogen, CA, USA) supplemented with 10% fetal bovine serum (Invitrogen) and incubated at 37 °C and 5% CO_2_. Commercial RNA interfering sets targeting LOC641518 were purchased from Ribobio technology (Shenzhen, Guangdong, China), and scramble siRNA was also transfected into breast cancer cells to regard it as control. Exogenous human full-length LEF1 plasmid vectors were purchased from Sino biological Inc. (Beijing, China); and empty vector was also transfected as negative control. Lipofectamine 3000 (Invitrogen) was used for all transfection studies by following the manufacturer’s protocol. Transfection efficiency was examined using qRT-PCR. Briefly, total RNA was extracted from cells using Trizol (Invitrogen, CA, USA) and further purified by RNAeasy mini kit plus DNase I treatment (Qiagen, Germany). The relative mRNA level for each gene was quantified by real-time RT-PCR with SYBR Green (Applied Biosystems, CA, USA), using GAPDH as a control.

### Wound healing assay

The migration ability of cells was measured by wound-healing assay. Briefly, 2 × 10^4^ cells were inoculated in 6-cm tissue culture dishes, cultured overnight, scratch was performed when cell growth fusion reached to 90%. Migration images were captured 24 and 48 h after scratching, the migration rate (width) of cells were calculated.

### Transwell migration

Corning™ BioCoat™ Matrigel™ Invasion Chamber with Corning™ Matrigel Matrix (Thermo Fisher Scientific, Waltham, MA, USA) was used for cell invasion assay. One hundred microliters of cells with concentration of 5 × 10^5^/mL were added in the upper chamber with (8 μm pore), 600 μL medium containing 10% serum was added in the lower chamber. After cultured for 6 h, medium in the chamber were removed and unmigrated cells were swabbed. Cells was fixed by 4% polyformaldehyde for 10 min, stained with crystal violet for another 10 min. The filter membrane was photographed under the inverted microscope (200×) after sealed with the neutral gum. Cells were counted by Image Pro Plus Version 6, three wells in each group and five vision fields of each well were randomly selected to calculate the average number of cells.

## Results

### Identification of DEmRNAs in tumor tissues from bone metastatic sites compared with other metastatic sites of breast cancer

**Fig. 1:**
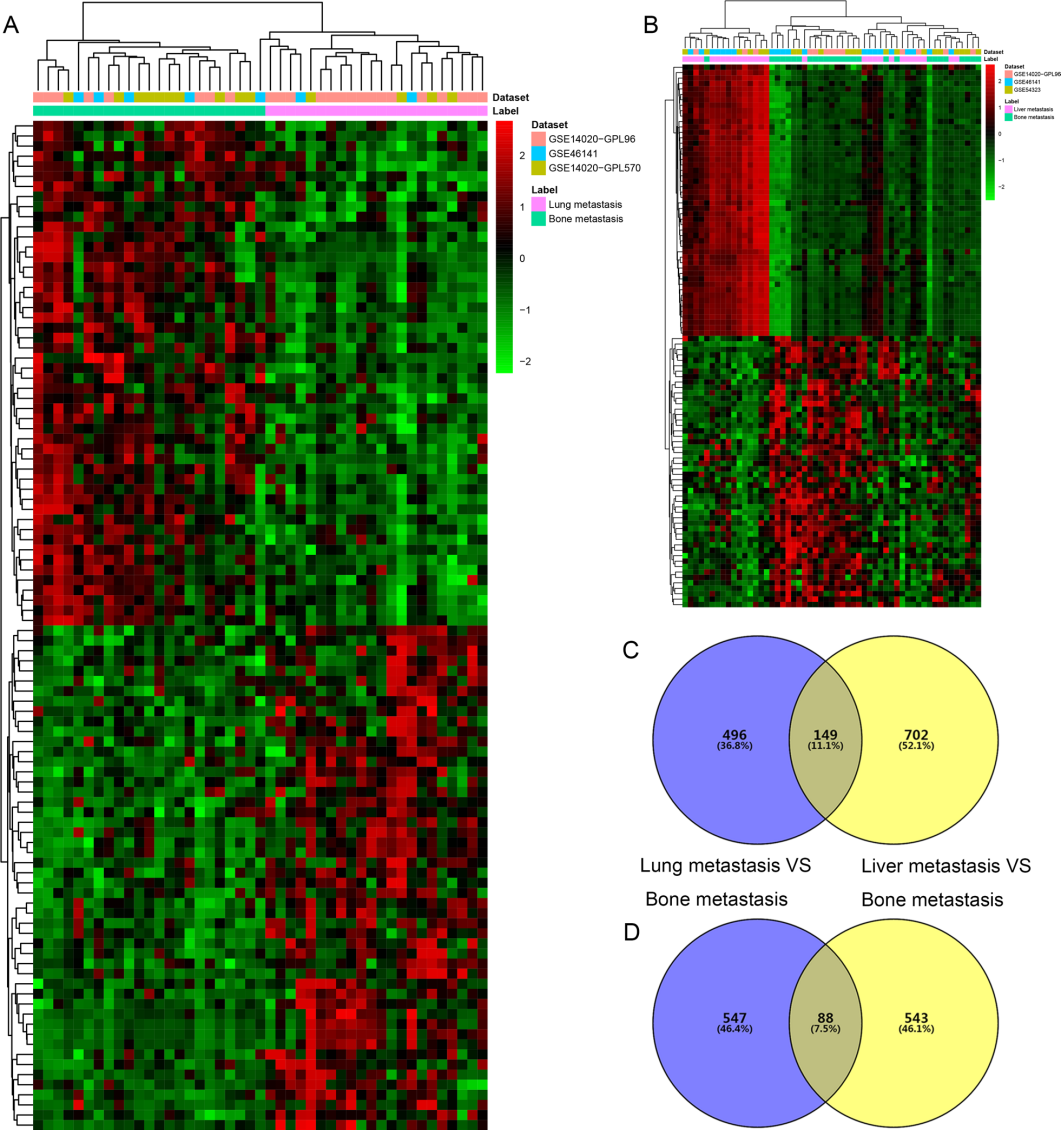
Identification of DEmRNAs. **A** Hierarchical clustering analysis of the top 100 DEmRNAs in tumor tissues from bone metastatic sites compared with lung metastatic sites of breast cancer. **B** Hierarchical clustering analysis of the top 100 DEmRNAs in tumor tissues from bone metastatic sites compared with liver metastatic sites of breast cancer. Row and column represented DEmRNAs and tissue samples, respectively. The color scale represented the expression levels. The red and green color represented the up- and down-regulated. **C** Venn diagram of up-regulated DEmRNAs in bone metastatic sites, lung metastatic sites and liver metastatic of breast cancer. **D** Venn diagram of down-regulated DEmRNAs in bone metastatic sites, lung metastatic sites and liver metastatic sites of breast cancer. Purple represented DEmRNAs in tumor tissues from bone metastatic sites compared with lung metastatic sites of breast cancer, and yellow represented DEmRNAs in tumor tissues from bone metastatic sites compared with liver metastatic sites of breast cancer

Compared with lung metastatic sites, a total of 1280 DEmRNAs (645 up- and 635 down-regulated mRNAs) were detected in tumor tissues from bone metastatic sites of breast cancer. Compared with liver metastatic sites, a total of 1482 DEmRNAs (851 up- and 631 down-regulated mRNAs) were detected in tumor tissues from bone metastatic sites of breast cancer. Hierarchical clustering analysis of top 100 up- and down-regulated DEmRNAs was exhibited in Fig. 1A, B, respectively. Top 10 up- and down-regulated DEmRNAs were displayed in Table 2. After overlapped these 1280 DEmRNAs and 1482 DEmRNAs, a total of 237 DEmRNAs (149 up- and 88 down-regulated mRNAs), which were differentially expressed in bone metastasis compared with both lung metastasis and liver metastasis, were obtained (Fig. 1C, D, Additional file 2: Table S2, Additional file 3: Table S3).

**Table 2:** Top 10 up- and down-regulated DEmRNAs/DElncRNAs in bone metastasis vs lung/liver metastasis

mRNA				lncRNA			
ID	Symbol	*p*-value	Regulation	ID	Symbol	*p*-value	Regulation
Bone metastasis vs lung metastasis							
25903	OLFML2B	4.24E−08	Up	284688	LOC284688	0.001117	Up
2042	EPHA3	6.89E−08	Up	100192378	LOC100192378	0.001644	Up
4322	MMP13	2.04E−07	Up	286411	RP1-177G6.2	0.001895	Up
7424	VEGFC	3.50E−07	Up	100505881	LOC100505881	0.002948	Up
57125	PLXDC1	3.59E−07	Up	220070	C11orf76	0.00331	Up
2200	FBN1	4.61E−07	Up	29034	CPS1-IT	0.003648	Up
1301	COL11A1	6.15E−07	Up	642852	LOC642852	0.00492	Up
2028	ENPEP	7.09E−07	Up	378938	MALAT1	0.005842	Up
1513	CTSK	7.73E−07	Up	100126793	GHRLOS	0.006006	Up
3381	IBSP	8.25E−07	Up	100289509	LOC100289509	0.006176	Up
6439	SFTPB	1.42E−08	Down	255480	LOC255480	1.05E-05	Down
6440	SFTPC	5.58E−08	Down	401491	FLJ35024	0.001445	Down
6441	SFTPD	8.85E−08	Down	643650	LOC643650	0.002133	Down
28959	TMEM176B	2.57E−07	Down	100132741	LOC100132741	0.002262	Down
91316	LOC91316	4.73E−07	Down	154822	LOC154822	0.00236	Down
54101	RIPK4	6.18E−07	Down	100131551	LOC100131551	0.002396	Down
91353	IGLL3P	1.22E−06	Down	100128511	LOC100128511	0.003006	Down
745	C11orf9	2.20E−06	Down	100505601	LOC100505601	0.00489	Down
718	C3	3.17E−06	Down	84847	MGC16075	0.005031	Down
3507	IGHM	4.77E−06	Down	339789	C2orf46	0.005971	Down
Bone metastasis vs liver metastasis							
4318	MMP9	2.77E−08	Up	255031	FLJ35390	0.002272	Up
5396	PRRX1	4.19E−08	Up	10984	KCNQ1OT1	0.002645	Up
1292	COL6A2	6.77E−08	Up	84815	MGC12916	0.003608	Up
1464	CSPG4	1.31E−07	Up	200197	C1orf126	0.007723	Up
1291	COL6A1	9.48E−07	Up	149830	PRNT	0.007785	Up
1749	DLX5	1.38E−06	Up	400999	FLJ42351	0.008556	Up
4322	MMP13	4.08E−06	Up	100131814	NCRNA00271	0.009051	Up
1301	COL11A1	5.77E−06	UP	255512	LOC255512	0.010037	Up
7431	VIM	7.59E−06	Up	641518	LOC641518	0.01096	Up
169611	OLFML2A	8.39E−06	Up	283547	LOC283547	0.011532	Up
2266	FGG	0	Down	140828	NCRNA00261	0.000205	Down
2244	FGB	3.11E−15	Down	100507008	LOC100507008	0.000512	Down
350	APOH	1.33E−14	Down	791114	PWRN1	0.001197	Down
2638	GC	5.75E−13	Down	283120	H19	0.002122	Down
2243	FGA	6.04E−13	Down	399806	FLJ41350	0.004141	Down
338	APOB	1.40E−12	Down	644246	LOC644246	0.004277	Down
259	AMBP	5.35E−12	Down	285972	LOC285972	0.004303	Down
1571	CYP2E1	5.65E−12	Down	255167	LOC255167	0.004445	Down
3273	HRG	8.57E−12	Down	149134	LOC149134	0.006425	Down
345	APOC3	1.18E−11	Down	619423	FAM85A	0.00673	Down

*DEmRNAs* differentially expressed mRNAs, *DElncRNAs* differentially expressed lncRNAs

### GO and KEGG pathway analysis of DEmRNAs

**Fig. 2:**
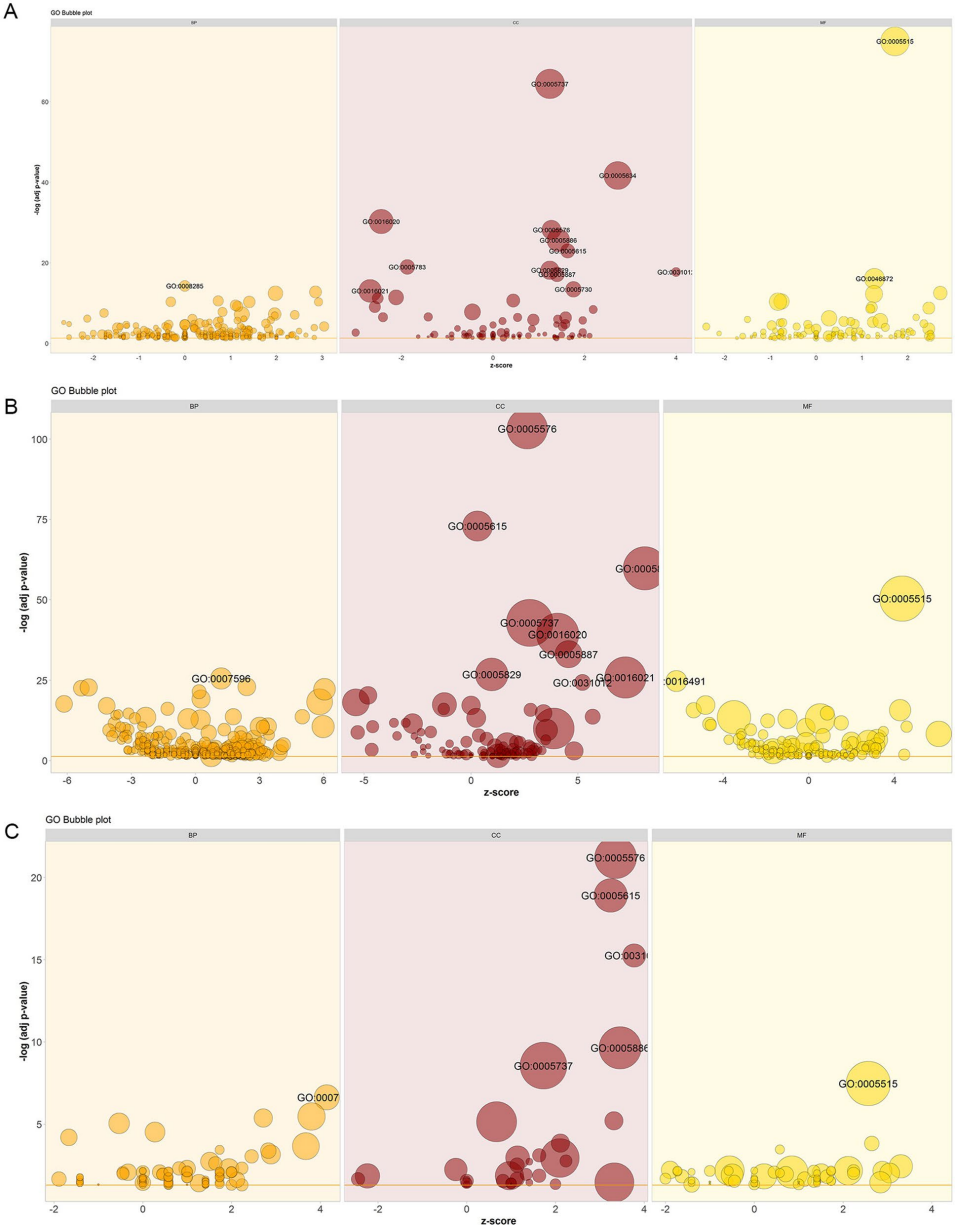
Significantly enriched GO terms of DEmRNAs in tumor tissues from bone metastatic sites compared with other metastatic sites of breast cancer. **A** Bone metastasis vs lung metastasis **B** Bone metastasis vs liver metastasis **C** Common DEmRNAs in bone metastasis vs lung metastasis and bone metastasis vs liver metastasis. The x-axis represented z-score and the y-axis represented -lg (adj *p*-value)

GO analysis indicated that DEmRNAs in group of bone metastasis vs lung metastasis were significantly enriched in negative regulation of cell proliferation (GO: 0008285; FDR = 5.43E−15), cytoplasm (GO: 0005737; FDR = 3.82E−65) and protein binding (GO: 0005515; FDR = 9.71E−76) (Fig. 2A); DEmRNAs in bone metastasis vs liver metastasis group were significantly enriched in blood coagulation (GO: 0007596; FDR = 2.81E−26), extracellular region (GO: 0005576; FDR = 5.82E−104) and protein binding (GO: 0005515; FDR = 4.33E−51) (Fig. 2B); the common DEmRNAs in two group mentioned above were significantly enriched in cell adhesion (GO: 0007155; FDR = 2.35E−07), extracellular region (GO: 0005576; FDR = 6.22E−22) and protein binding (GO: 0005515; FDR = 3.35E−08) (Fig. 2C), respectively. All the top 10 GO list between these two groups were shown in Additional file 4: Table S4.

**Fig. 3:**
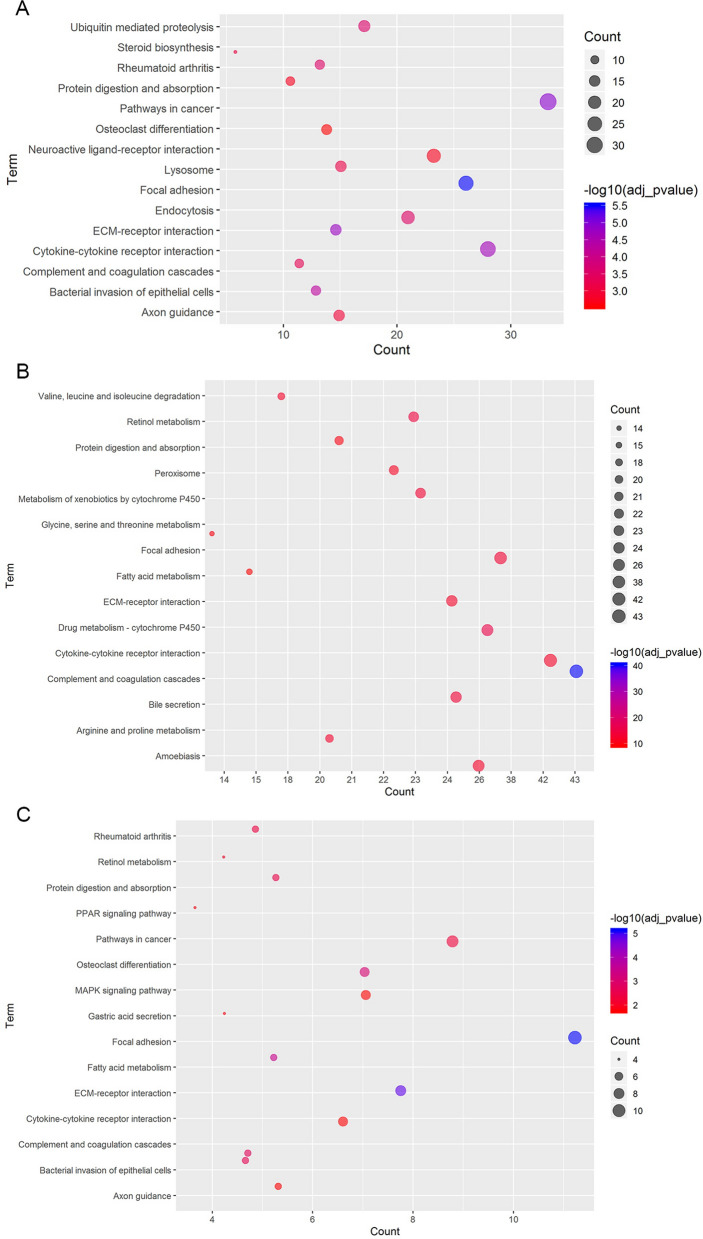
Significantly enriched KEGG pathways of DEmRNAs in tumor tissues from bone metastatic sites compared with other metastatic sites of breast cancer. **A** Bone metastasis vs lung metastasis; **B** Bone metastasis vs liver metastasis; **C** Common DEmRNAs in bone metastasis vs lung metastasis and bone metastasis vs liver metastasis. The x-axis shows counts of DEmRNAs enriched in KEGG pathways and the y-axis shows KEGG pathways. The color scale represented -lg (adj *p*-value)

KEGG pathway enrichment analysis indicated that DEmRNAs in group of bone metastasis vs lung metastasis were significantly enriched in Focal adhesion (FDR = 3.08E−06), Pathways in cancer (FDR = 1.22E−05) and ECM-receptor interaction (FDR = 1.73E−05) (Fig. 3A); DEmRNAs in bone metastasis vs liver metastasis group were significantly enriched in complement and coagulation cascades (FDR = 3.77E−41), Drug metabolism-cytochrome P450 (FDR = 2.63E−16) and Retinol metabolism (FDR = 1.07E−14) (Fig. 3B); the common DEmRNAs in two group mentioned above were significantly enriched in Focal adhesion (FDR = 7.32E−06), ECM-receptor interaction (FDR = 1.39E−05) and Fatty acid metabolism (FDR = 3.61E−04) (Fig. 3C), respectively.

### PPI network construction

**Fig. 4:**
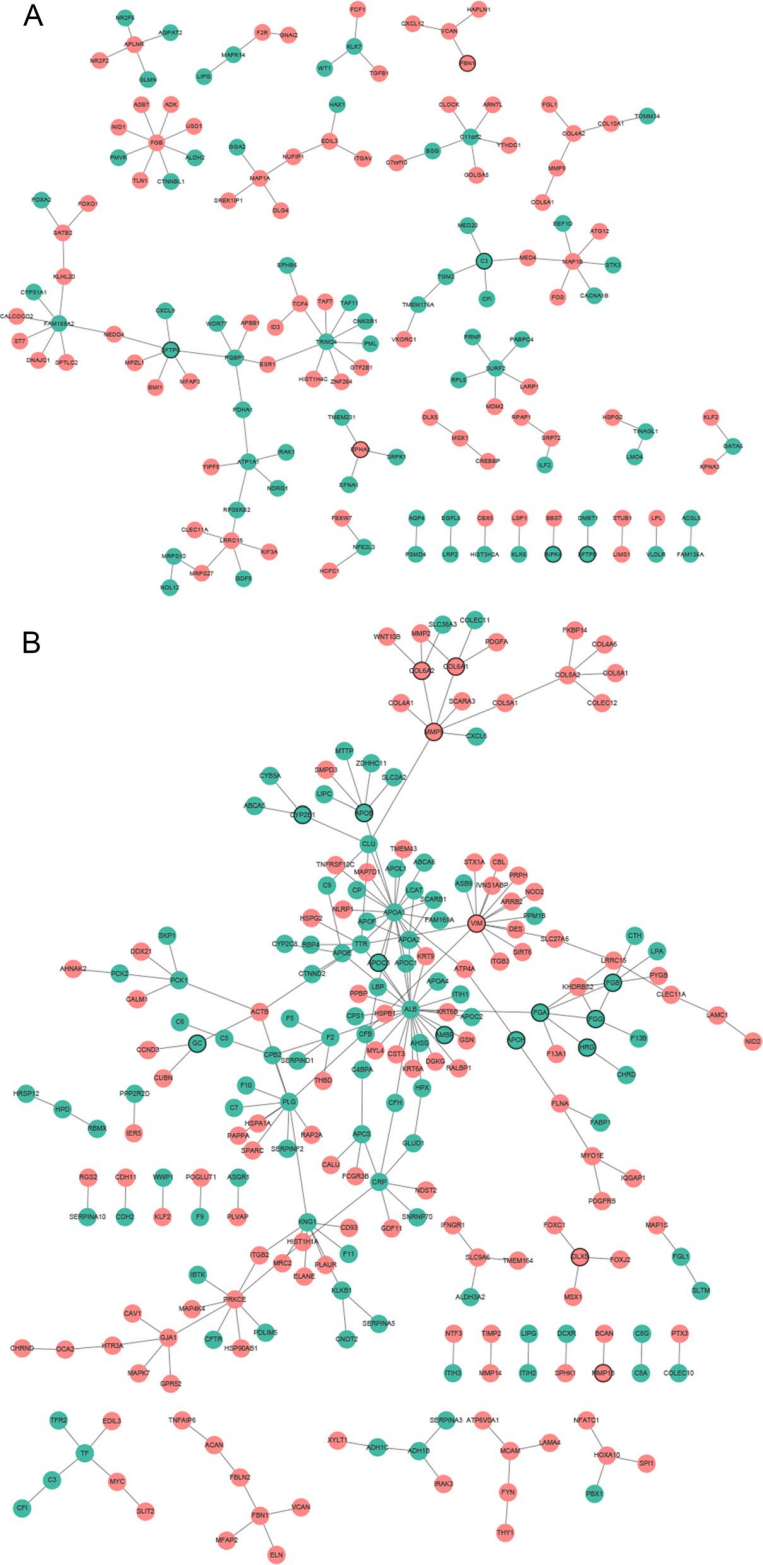
Protein–protein interaction (PPI) networks of DEmRNAs. **A** Bone metastasis vs lung metastasis; **B** Bone metastasis vs liver metastasis. The red and green circles represented proteins encoded by up- and down-regulated DEmRNAs. Circles with black border were DEmRNAs derived from top 10 down- and up-regulated DEmRNAs

The PPI network constructed with DEmRNAs in group of bone metastasis vs lung metastasis included 147 nodes and 121 edges. TRIM24 (degree = 9), FGB (degree = 8) and FAM189A2 (degree = 7) were three hub proteins of the PPI network (Fig. 4A). The PPI network constructed with DEmRNAs in group of bone metastasis vs liver metastasis included 231 nodes and 228 edges. TNFAIP6 (degree = 26), ACAN (degree = 21) and ADH1C (degree = 14) were three hub proteins of the PPI network (Fig. 4B).

### Identification of DElncRNAs in tumor tissues from bone metastatic sites compared with other metastatic sites of breast cancer

**Fig. 5:**
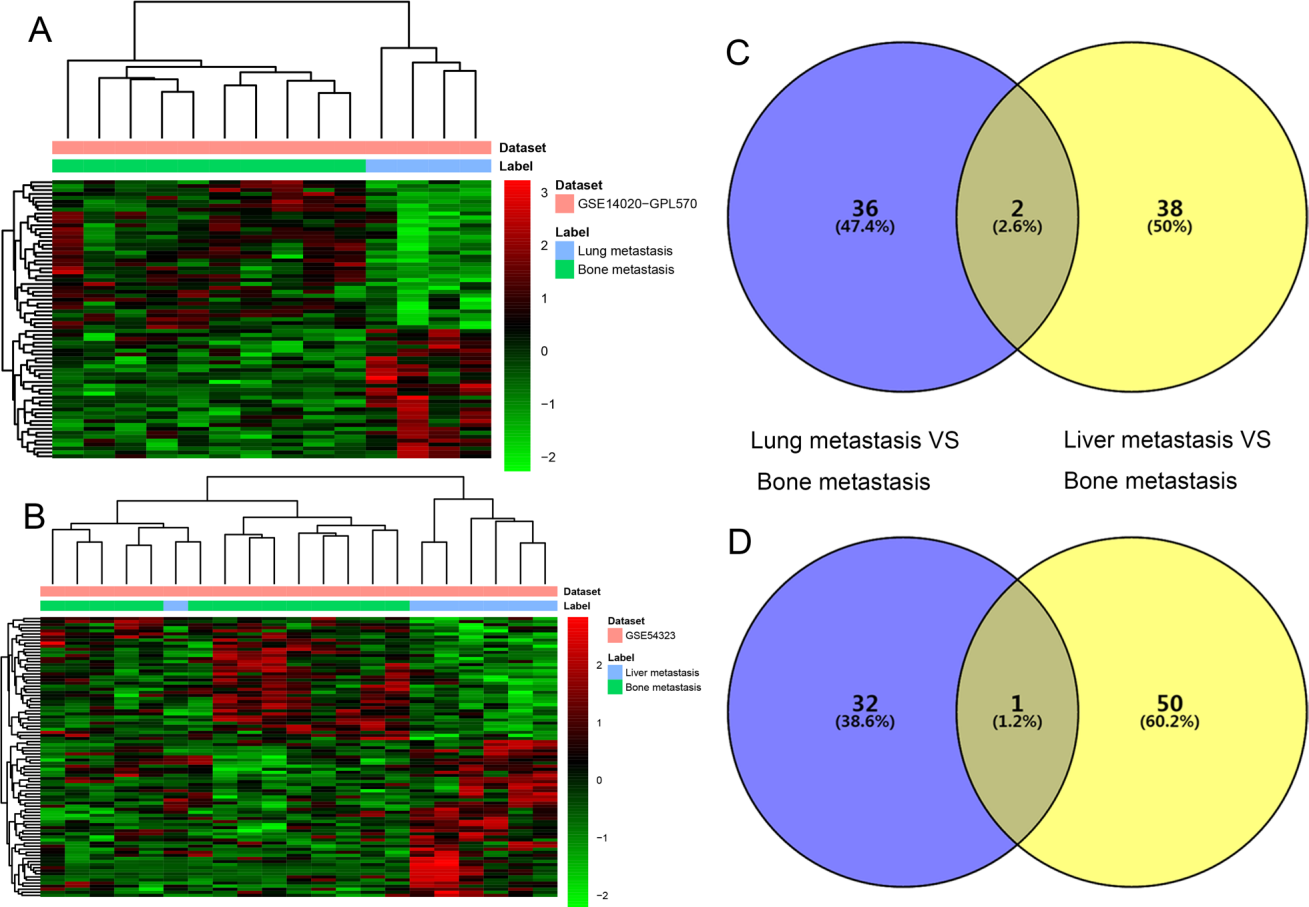
Identification of DElncRNAs. **A** Hierarchical clustering analysis of DElncRNAs in tumor tissues from bone metastatic sites compared with lung metastatic sites of breast cancer. **B** Hierarchical clustering analysis of DElncRNAs in tumor tissues from bone metastatic sites compared with liver metastatic sites of breast cancer. Row and column represented DElncRNAs and tissue samples, respectively. The color scale represented the expression levels. The red and green color represented the up- and down-regulated. **C** Venn diagram of up-regulated DElncRNAs in bone metastatic sites, lung metastatic sites and liver metastasis of breast cancer. **D** Venn diagram of down-regulated DElncRNAs in bone metastatic sites, lung metastatic sites and liver metastasis of breast cancer. Purple represented DElncRNAs in tumor tissues from bone metastatic sites compared with lung metastatic sites of breast cancer, and yellow represented DElncRNAs in tumor tissues from bone metastatic sites compared with liver metastatic sites of breast cancer

Compared with lung metastatic sites, a total of 71 DElncRNAs (38 up- and 33 down-regulated lncRNAs) were detected in tumor tissues from bone metastatic sites of breast cancer (Additional file 5: Table S5). Compared with liver metastatic sites, a total of 91 DElncRNAs (40 up- and 51 down-regulated lncRNAs) were detected in tumor tissues from bone metastatic sites of breast cancer (Additional file 6: Table S6). Hierarchical clustering analysis of DElncRNAs was exhibited in Fig. 5A, B, respectively. Top 10 up- and down-regulated DElncRNAs were displayed in Table 2. After overlapped these 71 DElncRNAs and 91 DElncRNAs, a total of three DElncRNAs (two up- and one down-regulated lncRNAs), which were differentially expressed in bone metastasis compared with both lung metastasis and liver metastasis, were obtained (Fig. 5C, D, Table 3).

**Table 3:** The common DElncRNAs in bone metastasis vs lung/liver metastasis

ID	Symbol	log_2_FC	*p*-value	Regulation
Bone metastasis vs lung metastasis				
641518	LOC641518	0.505116	0.012121	Up
127841	LOC127841	0.146866	0.038941	Up
285972	LOC285972	− 0.12832	0.043165	Down
Bone metastasis vs liver metastasis				
641518	LOC641518	0.204756	0.01096	Up
127841	LOC127841	0.163088	0.029473	Up
285972	LOC285972	− 0.21251	0.004303	Down

*DElncRNAs* differentially expressed lncRNAs, *FC* fold change

### Correlation study of DElncRNAs and DEmRNAs

The correlation study between DELncRNA and DEmRNA expression was analyzed by Person’s test, the correlation coefficient and p-value were shown in Table 4 (bone metastasis vs lung metastasis and bone metastasis vs liver metastasis), only pairs whose P value was less than 0.05 were shown.

**Table 4:** The correlation study between DElncRNAs and DEmRNAs in bone metastasis vs lung/liver metastasis

mRNA	lncRNA	Coefficient	P value
Bone metastasis vs lung metastasis			
CLCC1	LOC642864	0.63860351	0.013967298
VLDLR	LOC642864	0.541350812	0.045579809
CLCC1	LOC100131551	0.547584484	0.04267076
LEF1	LOC100131551	− 0.549536925	0.041788266
CLCC1	LOC641518	− 0.573563483	0.03199397
LEF1	LOC641518	0.73627139	0.00267603
CLCC1	LOC100505881	− 0.541529321	0.045494545
LEF1	LOC100505881	0.88603087	2.46E−05
CLCC1	FLJ35024	0.623038869	0.017302791
VLDLR	FLJ35024	0.846602729	0.000134025
LEF1	LOC285972	− 0.622215761	0.017494612
VLDLR	LOC255480	0.579893836	0.029724979
RARRES2	LOC255480	0.703951313	0.004954559
Bone metastasis vs liver metastasis			
TRAM2	PRNT	0.777508799	3.36E−05
HOXD10	PRNT	0.512329942	0.017570208
OR1F2P	PRNT	0.680044011	0.000694447
TNFRSF10C	PRNT	0.504008236	0.019825478
TRAM2	LOC730101	− 0.591048808	0.00477898
TNFRSF10C	LOC730101	− 0.518705367	0.015985969
HOXC13	HOTAIR	0.670342938	0.000883421
TNFRSF10C	HOTAIR	− 0.577130521	0.006157548
RARRES2	LOC200772	0.770322238	4.41E−05
TRAM2	LOC285972	− 0.61211127	0.003185745
HAND1	LOC285972	− 0.535398257	0.01237707
OR1F2P	LOC285972	− 0.595499878	0.004396446
KCNJ15	LOC283547	0.546337212	0.010394044
HOXC13	LOC283547	− 0.515360281	0.016802157
TNFRSF10C	LOC283547	0.592506067	0.004650814
KCNJ15	FLJ39639	0.501750419	0.020475731
TRAM2	FLJ39639	0.551831814	0.009500612
OR1F2P	FLJ39639	0.638119484	0.001854382
TNFRSF10C	FLJ39639	0.592733856	0.00463104
RASSF2	LOC254896	0.722097721	0.000218972
KCNJ15	LOC254896	0.688928451	0.00055273
TRAM2	LOC254896	0.593599355	0.004556544
HOXC13	LOC254896	− 0.661378297	0.001095227
OR1F2P	LOC254896	0.613681346	0.003087407
TNFRSF10C	LOC254896	0.823070336	4.61E−06

### *Cis* nearby-targeted DEmRNAs of the DElncRNAs

A total of seven DElncRNA-nearby-targeted DEmRNA pairs in group of bone metastasis vs lung metastasis, involving in seven DElncRNAs and seven DEmRNAs, were detected (Table 5). A total of 15 DElncRNA-nearby-targeted DEmRNA pairs in group of bone metastasis vs liver metastasis, involving in 12 DElncRNAs and 15 DEmRNAs, were detected (Table 5). Combined with correlation study, total 8 pairs were selected with significant correlation, and four pairs had correlation coefficient higher than 0.7, which were LOC641518-LEF1, FLJ35024-VLDLR, LOC285972-RARRES2 and LOC254896-TNFRSF10C respectively.

**Table 5:** Nearby targeted DEmRNAs of DElncRNAs in tumor tissues from bone metastatic sites compared with lung/liver metastatic sites of breast cancer

DElncRNA				Nearby targeted DEmRNA		
chr	Symbol	Start − 100 kb	End + 100 kb	Symbol	Start	End
Bone metastasis vs lung metastasis						
1	LOC642864	108757217	108958524	CLCC1	108929508	108963457
3	LOC100131551	194196465	194412803	LRRC15	194355247	194369743
4	LOC641518	108067525	108356836	LEF1	108047545	108168956
7	LOC100505881	79352877	79571208	MAGI2	78017057	79453574
9	FLJ35024	2321597	2743359	VLDLR	2621834	2660053
7	LOC285972	150333654	150548140	RARRES2	150338317	150341674
12	LOC255480	114308191	114512831	TBX5	114353931	114408442
Bone metastasis vs liver metastasis						
20	PRNT	4631282	4840668	PRND	4721910	4728460
20	PRNT	4631282	4840668	RASSF2	4780023	4823645
21	DSCR8	38021451	38256511	KCNJ15	38155549	38307357
21	DSCR8	38021451	38256511	KCNJ6	37607376	38121345
6	LOC730101	52564366	52769155	TRAM2	52497402	52576915
2	LOC401022	176064051	176288958	HOXD9	176122720	176124937
2	LOC401022	176064051	176288958	HOXD10	176108790	176119942
12	HOTAIR	53862308	54074956	HOXC13	53938765	53946544
7	LOC285972	150333654	150548140	RARRES2	150338317	150341674
5	FLJ38109	154229437	154545850	HAND1	154474972	154478264
14	LOC283547	38649339	39048273	SEC23A	39031919	39109646
1	LOC127841	204268431	204469719	PLEKHA6	204218851	204377665
16	FLJ39639	3163743	3367567	OR1F2P	3215611	3216543
8	LOC254896	22984355	23203558	TNFRSF10C	23102590	23117437

*DEmRNAs* differentially expressed mRNAs, *DElncRNAs* differentially expressed lncRNAs

### qRT-PCR validation

**Fig. 6:**
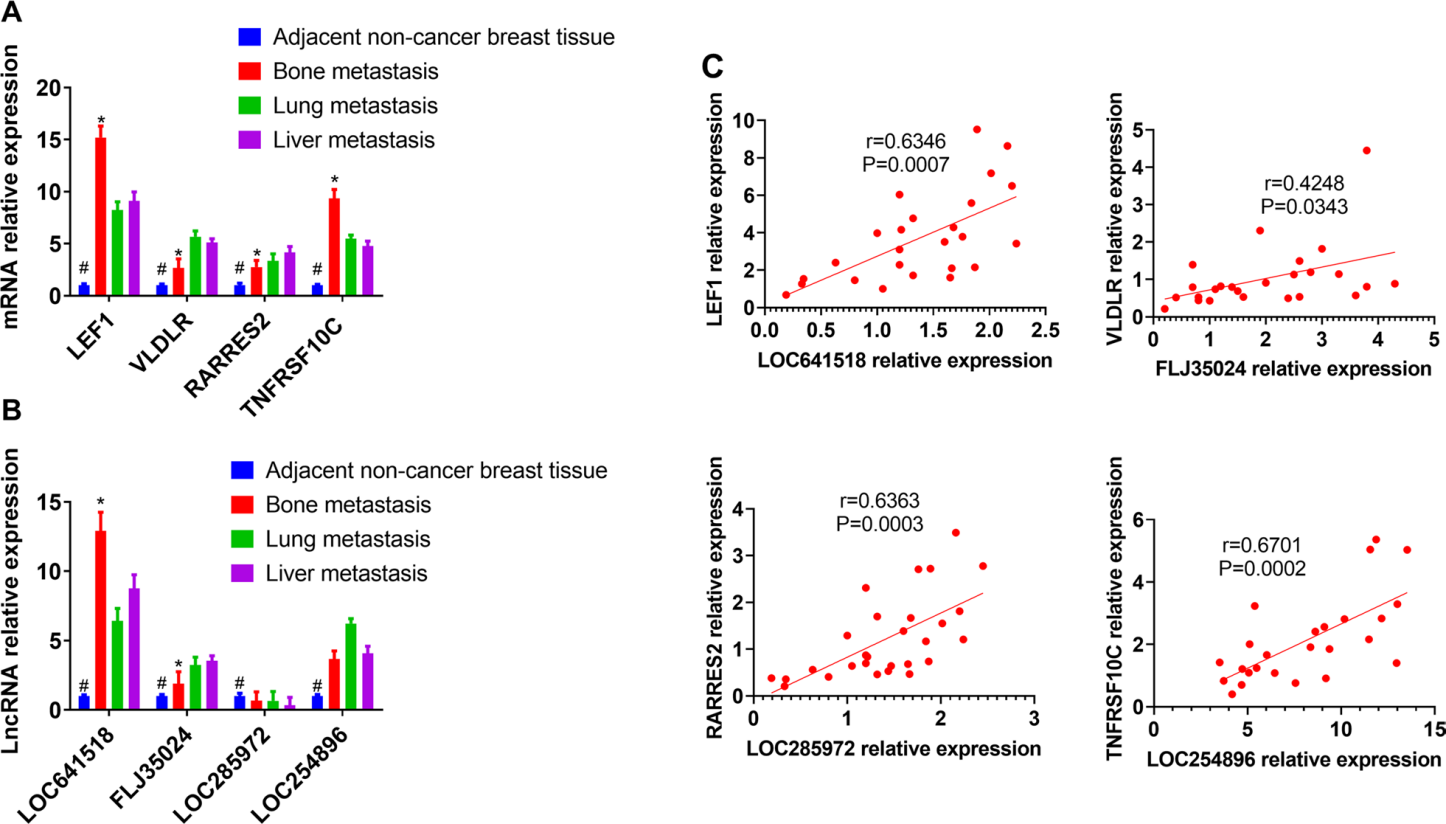
The qRT-PCR results of DEmRNAs and DElncRNAs in tumor tissues from bone metastatic sites compared with other metastatic sites of breast cancer. **A** Expression of LEF1, VLDLR, RARRES2 and TNFRSF10C in the tissue of patients with breast cancer bone metastasis, lung metastasis and liver metastasis by qRT-PCR. The x axis and y axis presented gene name and relative expression, respectively. **B** Expression of LOC641518, FLJ35024, LOC285972 and LOC254896 in the tissue of patients with breast cancer bone metastasis, lung metastasis, and liver metastasis by qRT-PCR. The x axis and y axis presented gene name and relative expression, respectively. **C** Dot-plot graphic of correlation between LOC641518-LEF1, FLJ35024-VLDLR, LOC200772-AGXT, LOC285972-RARRES2 and LOC254896-TNFRSF10C. #P < 0.05, adjacent non-tumor breast tissue compared with three site metastases; *p < 0.05, bone metastasis compared with lung and liver metastasis

These four pairs whose correlation coefficient was above 0.7 were selected for verification by qRT-PCR using clinical samples from our hospital. As shown in Fig. 6A, results showed that the relative expression of LEF1, VLDLR, RARRES2 and TNFRSF10C were significantly up-regulated compared to adjacent non-tumor tissues and other metastatic sites (P < 0.05). As shown in Fig. 6B, RNA levels of LncRNA LOC641518 and FLJ35024 showed same trend with bioinformatics data. Although RNA levels of LOC285972 and LOC254896 in the bone metastasis were also higher than adjacent non-tumor tissues, they were not different compared with lung and liver metastasis. All the mRNA and LncRNA levels of tested genes from adjacent non-tumor breast cancer was significantly lower than metastasis tumor tissues (Fig. 6). After that, we used the RNA relative levels of each pair LncRNA-mRNA from metastatic bone tumor tissues to perform a correlation analysis, which was shown in Fig. 6C. Results demonstrated all the four LncRNA pairs had significantly positive correlation, although the coefficient is not high as bioinformatics prediction. The validation test by qRCR proved that most of bioinformatics predictions were reliable.

### LOC641518 promotes breast cancer metastasis via activation LEF1

Due to LEF1 has been long considered as a metastasis mediator [14, 15], in the current study, the Loc641518-LEF1 axis was selected to investigate whether Loc641518 regulates LEF1 expression to promote breast cancer metastasis by in vitro study, which can help to confirm the speculation from bioinformatics data mining.

**Fig. 7:**
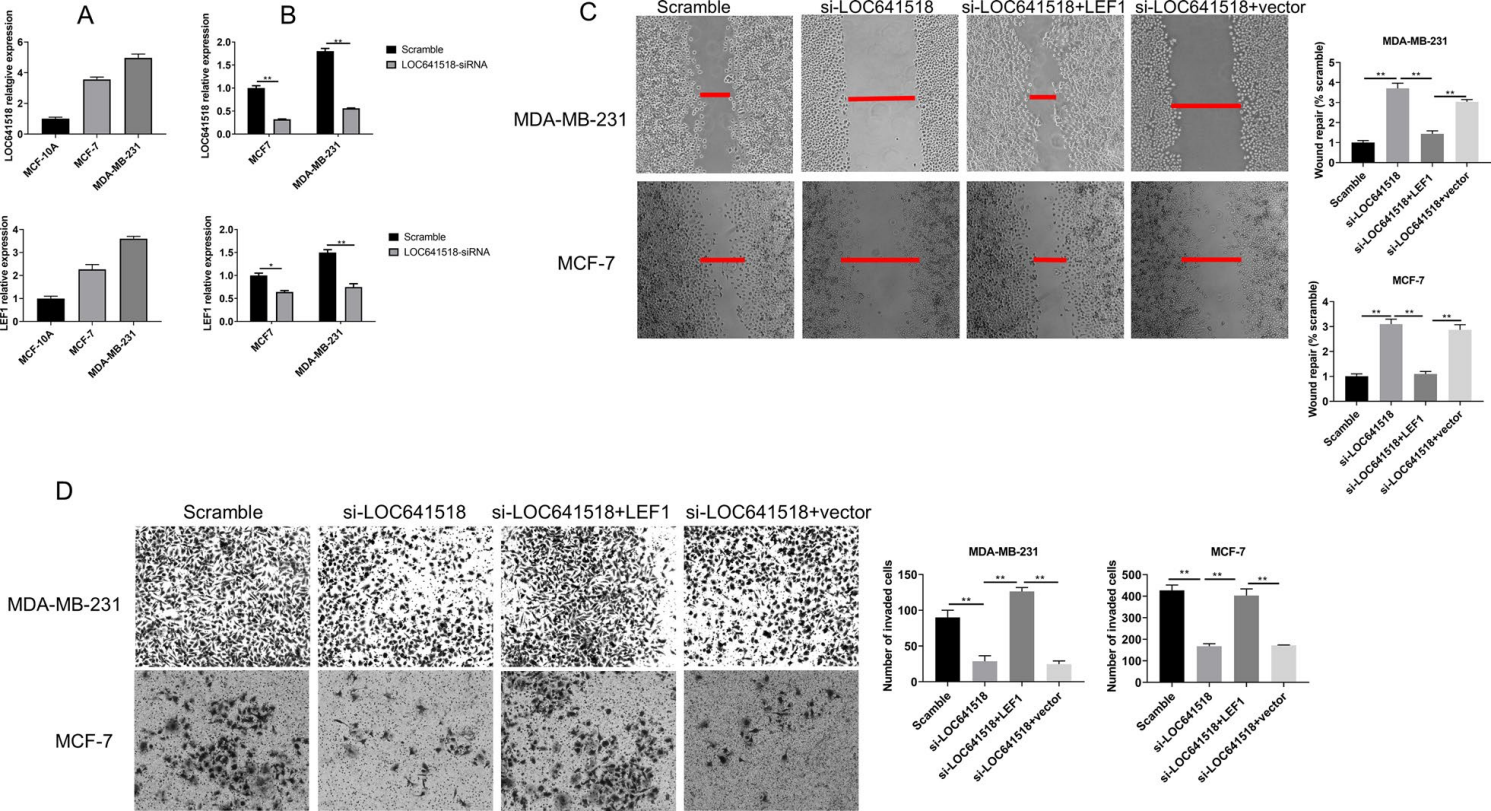
Wound healing assay and transwell invasion assay. **A** RT-PCR analysis of gene expression of LOC641518 and LEF1 in MCF-7 and MDA-MB-231 cells; **B** RT-PCR analysis of gene expression of LOC641518 and LEF1 in MCF-7 and MDA-MB-231 cells after knocking down LOC641518. **B** Wound closure was shown at 48 h post scratch (200×) after LOC641518 knocking down in MCF-7 and MDA-MB-231 cells, and over-expression LEF reverses its effect on wound closure. Cell migration was assessed by recover of the scratch. The area of the wound was measured at the two time points in every group, and % reduction of initial scratch area was compared with scramble; and the results were expressed as percentage relative to the corresponding scramble control. **C** Transwell invasion assay. MCF-7 and MDA-MB-231 cells penetrating the membrane were fixed and 0.1% crystal violet stained after 24 h as described in experimental procedures, and cell number penetrating the membrane were counted for each group. *P < 0.05, **P < 0.01. Data were the means of three measurements and the bars represented SD of the mean

The RNA levels of LOC641518 and LEF1 in non-carcinoma human breast epithelial cell line MCF-10A and two breast cancer cell lines, MCF-7 and MDA-MB-231, were examined by qRT-PCR (Fig. 7A, B), which could find that RNA levels of LOC641518 and LEF1 is higher in cancer cells than non-carcinoma cells, and highest in MDA-MB-231.

siRNA was used to knock down the expression of LOC641518 in both MCF7 and MDA-MB-231 cells. As shown in Fig. 7C, the expression of LOC641518 was significantly reduced by siRNA compared with scramble siRNA control. Meanwhile, the LEF1 mRNA level was also reduced in siRNA-LOC641518 groups compared with scramble groups (Fig. 7D). The wound healing assay and migration assay both demonstrated that knocking down LOC641518 significantly reduced capability of migration and invasion (Fig. 7E, F), and exogenous overexpression of LEF1 could reverse these phenomena.

## Discussion

Bone is a primary site for breast cancer metastases, and patients often suffer from osteolytic bone metastases, which is incurable [16]. Previously, we conducted integrated bioinformatics analysis to find several possible key genes which are related with bone metastasis [13]. Currently, we used same gene dataset, expanding to find differential LncRNAs which are associated with bone metastasis, and further elucidate more possible LncRNA-mRNA interaction pairs. The purpose of this study is to introduce a broaden vision that more complicated gene networks are involved into the bone metastasis of breast cancer.

The most merit of the current study is that 71 DElncRNAs (bone metastatic vs lung metastasis) and 91 DElncRNAs (bone metastasis vs liver metastasis) have been identified. By correlation study between these DElncRNAs and DEmRNAs, combined with *cis* nearby-targeted analysis, total nine LncRNA-mRNA interaction pairs were identified. By qRT-PCR study using clinical samples from our hospital, we confirm that most predictions are accurate and reliable. All these enriched data provide a landscape for researcher to clarify the role of LncRNA in breast cancer bone metastasis.

By knocking down the LOC641518, we observed reduced migration and invasion ability which was consistent with lower mRNA levels of LEF1, and over-expression of LOC641518 reversed its effect. LEF1, a member of the T cell Factor (TCF)/LEF1 family of high-mobility group transcription factors, is a downstream component of the Wnt signaling pathway [17]. LEF1 and HOXB9, two Wnt target genes, are identified as promoters of lung adenocarcinoma metastasis, and WNT/TCF pathway is identified as a determinant of metastasis to bone in lung adenocarcinoma [14]. In addition, it was reported that LEF1 was implicated in cell invasion and metastasis of breast cancer [18, 19]. LEF1 antisense RNA 1 (LEF1-AS1/LOC641518) is an antisense lncRNA encoded in the LEF1 locus, which is related to the metastasis in various tumors, including colorectal cancer and esophageal squamous cell carcinoma [20, 21]. Current study identified that LOC641518 *cis-*regulated the expression of LEF1 in group of bone metastasis vs lung metastasis, which might indicate that LOC641518 involved in breast cancer bone metastasis through regulating LEF1.

LOC285972-RARRES2, is another interaction pair identified in the current study. RARRES2, also known as chemerin, was considered as a chemoattractant and an adipokine [22]. The correlation between RARRES2 and cancers has been reported. Reduced expression of RARRES2 is observed in human hepatocellular carcinoma [23]. Lu et al. indicate RARRES2 that promotes tumorigenesis and metastasis in oral squamous cell carcinoma [24]. In addition, RARRES2 is identified as a potential biomarker for chordoma which is a rare, low-malignant bone tumor [25]. Low level of LINC00996 was report to be associated with colorectal carcinogenesis and metastasis [26]. In this study, LOC285972 was found to be down-regulated in both two groups and cis-regulate RARRES2 in the group of bone metastasis vs lung metastasis. Hence, we hypothesize that LOC285972-RARRES2 interaction pair is associated with the development of breast cancer bone metastasis.

Very low density lipoprotein receptor (VLDLR) has been considered as a multiple function receptor due to binding numerous ligands, causing endocytosis and regulating cellular signaling [27, 28]. Previous studies have confirmed that high VLDLR expression is correlated with lymph node and distant metastasis in gastric and breast cancer patients, that VLDLR may be a clinical marker in cancers, and has a potential link with β-catenin signaling pathway [27]. In the current study, we predict that FLJ35024 cis-regulate VLDLR, but very rare report about FLJ35024 so far. Therefore, their interaction deserves further study.

TNFRSF10C copy number variation has been reported that is associated with metastatic colorectal cancer [29], and its hypermethylation was associated with NSCLC [30]. One publication reports that TNFRSF10C plays a role in triple-negative breast cancer cell survival and metastasis regulated by SOX9 [31]. LOC254896 was expected to cis-regulate its expression by present bioinformatics analysis, but LOC254896 is still mysterious and lack of detailed study.

One of limitation is that we only verify the role of LOC641518-LEF1 interaction pair in migration and invasion capability of breast cancer cells, and the remaining interaction pairs also deserve further investigation. The other limitation is that there is no in vivo data to be provided in the current study, further, the experimental bone metastasis model should be established to investigate their role in mice. These findings may make contribution to understand the process of bone metastasis through lncRNA regulation, and further be helpful to develop new strategies that effectively predict occurrence of bone metastasis, also provide novel targets for drug design in the future clinical practice.

## Conclusion

In conclusion, four lncRNA-mRNA interaction pairs were identified to be closely associated with breast cancer bone metastasis in this analysis, and Loc641518-LEF1 axis was confirmed by in vitro study. All these results may provide the novel and additional therapeutic strategies for breast bone metastatic patients.

## Supplementary Information

**Additional file 1: Table S1.** Primer sequence

**Additional file 2: Table S2.** Differential expressed mRNAs detected in metastatic tumor tissues of breast cancer between bone and lung metastatic sites (bone vs lung).

**Additional file 3: Table S3.** Differential expressed mRNAs detected in metastatic tumor tissues of breast cancer between bone and liver metastatic sites (bone vs liver).

**Additional file 4: Table S4.** A: Top 10 Gene oncology list enriched by differential expressed mRNAs detected in metastatic tumor tissues of breast cancer between bone and lung metastatic sites; B: Top 10 Gene oncology list enriched by differential expressed mRNAs detected in metastatic tumor tissues of breast cancer between bone and liver metastatic sites; C: Top 10 overlapped gene oncology between GO list (bone metastasis vs lung metastasis) and GO list (bone metastasis vs liver metastasis).

**Additional file 5: Table S5.** Differential expressed LncRNAs detected in metastatic tumor tissues of breast cancer between bone and lung metastatic sites (bone vs lung).

**Additional file 6: Table S6.** Differential expressed LncRNAs detected in metastatic tumor tissues of breast cancer between bone and lung metastatic sites (bone vs lung).

## Data Availability

All the data are available on requirement.

## Abbreviations

lncRNAs: Long non-coding RNAs
qRT-PCR: Quantitative real-time polymerase chain reaction
GEO: Gene expression omnibus
DEmRNAs: Differentially expressed mRNAs
DELncRNAs: Differentially expressed LncRNAs
KEGG: Kyoto Encyclopedia of Genes and Genomes
GO: Gene ontology
LEF1: Lymphoid enhancer-binding factor 1
VLDLR: Very low density lipoprotein receptor
RARRES2: Retinoic acid receptor responder 2
TNFRSF10C: TNF receptor superfamily member 10c
MALAT1: Metastasis associated lung adenocarcinoma transcript 1
PPI: Protein–protein interaction
FDR: False discover rate

## References

[1] Zulauf N, Bruggmann D, Groneberg D, Oremek GM (2019). Expressiveness of bone markers in breast cancer with bone metastases. Oncology.

[2] Holen I, Lefley DV, Francis SE, Rennicks S, Bradbury S, Coleman RE, Ottewell P (2016). IL-1 drives breast cancer growth and bone metastasis in vivo. Oncotarget.

[3] Parkes A, Warneke CL, Clifton K, Al-Awadhi A, Oke O, Pestana RC, Alhalabi O, Litton JK, Hortobagyi GN (2018). Prognostic factors in patients with metastatic breast cancer with bone-only metastases. Oncologist.

[4] Brook N, Brook E, Dharmarajan A, Dass CR, Chan A (2018). Breast cancer bone metastases: pathogenesis and therapeutic targets. Int J Biochem Cell Biol.

[5] Wang Y, Wu N, Liu J, Wu Z, Dong D (2015). FusionCancer: a database of cancer fusion genes derived from RNA-seq data. Diagn Pathol.

[6] Chen Y, Yu X, Xu Y, Shen H (2017). Identification of dysregulated lncRNAs profiling and metastasis-associated lncRNAs in colorectal cancer by genome-wide analysis. Cancer Med.

[7] Gooding AJ, Zhang B, Jahanbani FK, Gilmore HL, Chang JC, Valadkhan S, Schiemann WP (2017). The lncRNA BORG drives breast cancer metastasis and disease recurrence. Sci Rep.

[8] Kim J, Piao HL, Kim BJ, Yao F, Han Z, Wang Y, Xiao Z, Siverly AN, Lawhon SE, Ton BN (2018). Long noncoding RNA MALAT1 suppresses breast cancer metastasis. Nat Genet.

[9] Li W, Jia G, Qu Y, Du Q, Liu B, Liu B (2017). Long non-coding RNA (LncRNA) HOXA11-AS promotes breast cancer invasion and metastasis by regulating epithelial-mesenchymal transition. Med Sci Monit.

[10] Li C, Wang S, Xing Z, Lin A, Liang K, Song J, Hu Q, Yao J, Chen Z, Park PK (2017). A ROR1-HER3-lncRNA signalling axis modulates the Hippo-YAP pathway to regulate bone metastasis. Nat Cell Biol.

[11] Marot G, Foulley JL, Mayer CD, Jaffrezic F (2009). Moderated effect size and P-value combinations for microarray meta-analyses. Bioinformatics.

[12] Ritchie ME, Phipson B, Wu D, Hu Y, Law CW, Shi W, Smyth GK (2015). limma powers differential expression analyses for RNA-sequencing and microarray studies. Nucleic Acids Res.

[13] Zhang Y, He W, Zhang S (2019). Seeking for correlative genes and signaling pathways with bone metastasis from breast cancer by integrated analysis. Front Oncol.

[14] Nguyen DX, Chiang AC, Zhang XH, Kim JY, Kris MG, Ladanyi M, Gerald WL, Massague J (2009). WNT/TCF signaling through LEF1 and HOXB9 mediates lung adenocarcinoma metastasis. Cell.

[15] Min RQ, Ma Q (2020). MicroRNA-381 inhibits metastasis and epithelial-mesenchymal transition of glioblastoma cells through targeting LEF1. Eur Rev Med Pharmacol Sci.

[16] Taipaleenmaki H, Farina NH, van Wijnen AJ, Stein JL, Hesse E, Stein GS, Lian JB (2016). Antagonizing miR-218-5p attenuates Wnt signaling and reduces metastatic bone disease of triple negative breast cancer cells. Oncotarget.

[17] Cadigan KM, Waterman ML (2012). TCF/LEFs and Wnt signaling in the nucleus. Cold Spring Harb Perspect Biol.

[18] Nguyen A, Rosner A, Milovanovic T, Hope C, Planutis K, Saha B, Chaiwun B, Lin F, Imam SA, Marsh JL (2005). Wnt pathway component LEF1 mediates tumor cell invasion and is expressed in human and murine breast cancers lacking ErbB2 (her-2/neu) overexpression. Int J Oncol.

[19] Zheng T, Wang A, Hu D, Wang Y (2017). Molecular mechanisms of breast cancer metastasis by gene expression profile analysis. Mol Med Rep.

[20] Shi Q, He Y, Zhang X, Li J, Cui G, Zhang X, Wang X (2019). Two novel long noncoding RNAs—RP11-296E3.2 and LEF1-AS1can—separately serve as diagnostic and prognostic bio-markers of metastasis in colorectal cancer. Med Sci Monit.

[21] Zong MZ, Feng WT, Du N, Yu XJ, Yu WY (2019). Upregulation of long noncoding RNA LEF1-AS1 predicts a poor prognosis in patients with esophageal squamous cell carcinoma. Eur Rev Med Pharmacol Sci.

[22] Ernst MC, Sinal CJ (2010). Chemerin: at the crossroads of inflammation and obesity. Trends Endocrinol Metab.

[23] Lin W, Chen YL, Jiang L, Chen JK (2011). Reduced expression of chemerin is associated with a poor prognosis and a lowed infiltration of both dendritic cells and natural killer cells in human hepatocellular carcinoma. Clin Lab.

[24] Lu Z, Liang J, He Q, Wan Q, Hou J, Lian K, Wang A (2019). The serum biomarker chemerin promotes tumorigenesis and metastasis in oral squamous cell carcinoma. Clin Sci.

[25] Scheil-Bertram S, Kappler R, von Baer A, Hartwig E, Sarkar M, Serra M, Bruderlein S, Westhoff B, Melzner I, Bassaly B (2014). Molecular profiling of chordoma. Int J Oncol.

[26] Ge H, Yan Y, Wu D, Huang Y, Tian F (2018). Potential role of LINC00996 in colorectal cancer: a study based on data mining and bioinformatics. Onco Targets Ther.

[27] He L, Lu Y, Wang P, Zhang J, Yin C, Qu S (2010). Up-regulated expression of type II very low density lipoprotein receptor correlates with cancer metastasis and has a potential link to beta-catenin in different cancers. BMC Cancer.

[28] Takahashi S, Kawarabayasi Y, Nakai T, Sakai J, Yamamoto T (1992). Rabbit very low density lipoprotein receptor: a low density lipoprotein receptor-like protein with distinct ligand specificity. Proc Natl Acad Sci USA.

[29] Tanenbaum DG, Hall WA, Colbert LE, Bastien AJ, Brat DJ, Kong J, Kim S, Dwivedi B, Kowalski J, Landry JC (2016). TNFRSF10C copy number variation is associated with metastatic colorectal cancer. J Gastrointest Oncol.

[30] Qi Y, Qi L, Qiu M, Yao C, Zhang M, Lin J, Zheng Z, Chen C, Li H, Duan S (2020). Hypermethylation of tumor necrosis factor decoy receptor gene in non-small cell lung cancer. Oncol Lett.

[31] Ma Y, Shepherd J, Zhao D, Bollu LR, Tahaney WM, Hill J, Zhang Y, Mazumdar A, Brown PH (2020). SOX9 is essential for triple-negative breast cancer cell survival and metastasis. Mol Cancer Res.

