# Supplementary material for: New insight into long non-coding RNAs associated with bone metastasis of breast cancer based on an integrated analysis
Source: Cancer Cell Int. 2021 Jul 13;21:372. doi: 10.1186/s12935-021-02068-7 (PMC8276423; doi:10.1186/s12935-021-02068-7)
Supplement: Supplementary file 1 — Additional file 1: Table S1. Primer sequence [file 12935_2021_2068_MOESM1_ESM.docx]

| Gene | Primer sequence (5’-3’) |
| --- | --- |
| LEF1-F | GAACCTCGTGGGGACAATTAC |
| LEF-R | CATCATAGCCATCGTAGCCTTG |
| VLDLR-F | CTGCAGGGACTGGAGTGATGAG |
| VLDLR-R | GCAGATTCCTGGATTTTGGCA |
| RARRES2-F | TGAGGACCCCCACAGCTTCT |
| RARRES2-R | AGGCACCACGCATCTCAGTG |
| TNFRSF10C-F | AGGGTGCGATTTAGGATTTAG |
| TNFRSF10C-R | CGATAACGACGACGAACTT |
| LOC641518-F | TGGGACCGTGAAAAAGTCGT |
| LOC641518-R | ACTGCAAGAAGGTTGTGGCT |
| FLJ35024-F | AGGAGCAGAAATCTTACCAGCA |
| FLJ35024-R | TTCAGGGGCAAGATGACCTG |
| LOC285972-F | CTCCCATCTTTTCTGCCGGT |
| LOC285972-R | AGGGGCATCCAAAGGTCTTG |
| LOC254896-F | CAGGACGAACTCGCCGTC |
| LOC254896-R | AACCGCTCTGTGTCTTCAGG |

Additional file 1:Table S1. Primer sequence
